# Sociodemographic factors associated with health-related quality of life in UK healthcare workers: a cross-sectional study

**DOI:** 10.1186/s12916-025-04208-6

**Published:** 2025-07-22

**Authors:** Christopher A. Martin, Rebecca F. Baggaley, Lucy Teece, Daniel Pan, Joshua Nazareth, Luke Bryant, Carol Rivas, Katherine Woolf, Manish Pareek, The UK-REACH study collaborative group, Katherine Woolf, Manish Pareek, Laura Gray, Laura Nellums, Anna L Guyatt, Catherine John, I Chris McManus, Ibrahim Abubakar, Amit Gupta, Avinash Aujayeb, Bindu Gregary, Rubina Reza, Sandra Simpson, Stephen Zingwe, Keith R Abrams, Martin D Tobin, Louise Wain, Sue Carr, Edward Dove, Kamlesh Khunti, David Ford, Robert Free

**Affiliations:** 1 Department of Respiratory Sciences, University of Leicester, Maurice Shock Medical Sciences Building, University Road, Leicester, LE1 9HN UK; 2 Development Centre for Population Health, University of Leicester, Maurice Shock Medical Sciences Building, University Road, Leicester, LE1 9HN UK; 3 Department of Infection and HIV Medicine, University Hospitals of Leicester NHS Trust, Leicester, LE1 7RH UK; 4 NIHR Leicester Biomedical Research Centre (BRC), George Davies Centre, University of Leicester, 15 Lancaster Rd, Leicester, LE1 7HA UK; 5 Department of Population Health Sciences, University of Leicester, George Davies Centre, 15 Lancaster Rd, Leicester, LE1 7HA UK; 6 Li Ka Shing Centre for Health Information and Discovery, Oxford Big Data Institute, University of Oxford, Old Road Campus, Oxford, OX3 7LF UK; 7 WHO Collaborating Centre for Infectious Disease Epidemiology and Control, School of Public Health, Faculty of Medicine, University of Hong Kong, Li Ka Sing, Hong Kong, China; 8 UCL Social Research Institute, University College London, 55-59 Gordon Square, London, WC1H 0NU UK; 9 UCL Medical School, University College London, 40 Bernard Street, Level 3, London, WC1N 1LE UK; 10 NIHR Applied Research Collaboration East Midlands, University of Leicester, Leicester, UK

**Keywords:** EQ-5D, Quality of life, Healthcare workers, Occupation, Deprivation, United Kingdom, Anxiety, Depression, Pain

## Abstract

**Background:**

Healthcare workers’ (HCW) health and wellbeing directly affect patient care, yet little is known about their health-related quality of life (HRQoL) in the United Kingdom (UK). Using data from a nationwide study conducted during the COVID-19 pandemic in the UK (December 2020 to March 2021), we evaluated self-reported HRQoL among HCWs and its variation by sociodemographic characteristics.

**Methods:**

HRQoL was measured using the five-dimension-five-level EuroQoL (EQ-5D-5L) questionnaire, covering five health dimensions. We explored differences in reported HRQoL by age, sex, Index of Multiple Deprivation (IMD) quintile, ethnicity, migration status and occupational group. Each HRQoL dimension was collapsed into a binary outcome (any problems versus none) and associations analysed using logistic regression. We also examined EQ-5D-5L visual analogue scale (VAS) scores using linear regression.

**Results:**

Among 12,026 HCWs, 75.9% were female, 26.7% overseas-born and 29.9% from non-White ethnic groups. HCWs reported high levels of pain/discomfort and anxiety/depression (43.7% and 46.1% reporting at least slight problems, respectively). Women were more likely than men to report problems across all EQ-5D-5L dimensions. Reporting health problems increased with increased deprivation. Asian overseas-born and Black HCWs were less likely than White UK-born HCWs to report anxiety/depression. Compared to the Medical group, other HCW types reported more problems with pain/discomfort (32.2% Medical, 55.4% Nursing, 45.4% Allied Health Professionals, 49.8% Ambulance groups) and anxiety/depression (36.8% Medical, 52.9% Nursing, 50.5% Ambulance groups). Nurses and ambulance workers showed particularly high rates of pain/discomfort. Overall, all HCWs reported more problems with anxiety/depression, usual activities and pain/discomfort than the Medical group. Similar associations were demonstrated in a parallel analysis of VAS scores.

**Conclusions:**

In the largest study of HRQoL in HCWs to date, EQ-5D-5L VAS scores were lower than those reported elsewhere for the general UK population (for ages up 45 years), with high levels of anxiety/depression and pain/discomfort and substantial heterogeneities across EQ-5D-5L dimensions by sex, occupation and deprivation level. However, HCWs’ circumstances during the COVID-19 pandemic may have influenced their reporting of HRQoL. Our findings highlight the need for further research to understand the causes of lower HRQoL, particularly among women and certain occupational groups, and to inform targeted interventions.

**Supplementary Information:**

The online version contains supplementary material available at 10.1186/s12916-025-04208-6.

## Background

Nearly two million people in the UK are employed in the healthcare workforce [1]. The health of healthcare workers (HCWs) is critical not only for the wellbeing of such a large proportion of the population, but also for their ability to render good quality services to their patients and for future service planning. Low health-related quality of life (HRQoL) may compromise quality of patient care through increased staff presenteeism, absenteeism and attrition, leading to understaffing, additional workload for other staff and longer waiting lists, with the attendant impact on the UK economy [2]. At an organisational level, HCW sickness absence is associated with a financial cost through paid sick leave and covering rota gaps with locum staff [3]. In the UK, this problem is compounded by the current NHS staffing crisis, with estimated shortages of 12,000 hospital doctors and over 50,000 nurses and midwives [4].

As a result, the UK DHSC has included improving health outcomes through a well-supported workforce as a priority outcome [5, 6] and there is a new emphasis on improving staff wellbeing [7, 8]. However, little has been reported about the HRQoL of HCWs in the UK, with the majority of recent research focussing on stress, burnout and attrition rather than the full spectrum of physical and mental health effects [9, 10]. We therefore analysed baseline data from the United Kingdom Research study into Ethnicity and COVID-19 outcomes in Healthcare workers (UK-REACH) cohort study, which is among the largest UK HCW cohort studies, to evaluate the HRQoL of the UK healthcare workforce. We aimed to identify any patterns in reporting lower HRQoL across the five identified dimensions of the EQ-5D-5L instrument, a questionnaire which measures HRQoL according to whether the respondent has problems with any of the following aspects: anxiety/depression; carrying out usual activities (e.g. work, study, housework, family or leisure activities); pain/discomfort; mobility and self-care. We also explored the association between reported HRQoL and sociodemographic factors including sex, age, occupation, ethnicity and migration status. Our analysis involved no hypothesis testing because our study aim was to explore a range of sociodemographic factors which may potentially be associated with HCWs’ perceptions of their own health.

## Methods

### Overview

UK-REACH is a programme of work established to investigate the disproportionate impact of the COVID-19 pandemic on HCWs from ethnic minority groups. For the present secondary data analysis, we used data collected in the baseline questionnaire of the UK-REACH prospective nationwide cohort study. The questionnaire was electronically administered between December 2020 and March 2021. For a full description of the study methods and the cohort, see the study cohort paper [11]. No personally identifiable information was collected in the questionnaire. Details of the measures included in the questionnaire can be found in the data dictionary (https://www.uk-reach.org/main/data-dictionary/).

The study was approved by the Health Research Authority (Brighton and Sussex Research Ethics Committee; ethics reference: 20/HRA/4718) and registered with ISRCTN (Reference ISRCTN 11811602).

### Study population and recruitment

We included HCWs (including ancillary workers in a healthcare setting) aged 16 years or older or registered with one of seven professional healthcare regulators, with registrants from across the four UK countries. The seven regulators issued emails and newsletters to their registrants containing a link to the study website. Recipients had to read the email, navigate to the study website and register to create a study profile. Following creation of their profile, potential participants were asked to read a participant information sheet and, if willing, to consent to participate in the study*.* The sample was supplemented with direct recruitment from participating NHS trusts. Further information regarding the recruitment process is detailed elsewhere [11, 12]. Participant response rates at each stage of the recruitment process were recorded as recommended by the Checklist for Reporting Results of Internet E-Surveys (CHERRIES) [13].

### Outcome measures

The EuroQoL five-dimension (EQ-5D) questionnaire is a versatile HRQoL instrument comprising a short descriptive system questionnaire covering five health dimensions (anxiety/depression; usual activities (e.g. work, study, housework, family or leisure activities); pain/discomfort; mobility and self-care) and a visual analogue scale (VAS). The questionnaire provides a simple descriptive profile of a respondent’s health state and aids decision-making within healthcare with its varied applications, including allowing for the calculation of quality adjusted life years [14, 15]. It is one of the most popular instruments for assessing HRQoL, having been validated in many languages and health conditions [16–18].

We used the five-level EQ-5D version (EQ-5D-5L [19, 20]), which has been validated for a wide range of disease states. The respondent selects one of five levels of severity for each of the five health dimensions (no problems, slight problems, moderate problems, severe problems, unable to/extreme problems). We conducted preliminary analysis using this five-point Likert scale. However, our study sample is composed of an active, working population, meaning that frequency of reporting problems on the EQ-5D-5L dimensions, particularly the self-care dimension, was low. We therefore explored differences between respondents by using a binary outcome (any versus no problems) for each EQ-5D-5L dimension and chose to focus our analysis on the EQ-5D-5L VAS as this continuous outcome more easily allowed us to identify differences and patterns in reporting by sociodemographic groups. We chose to present outcomes for each EQ-5D-5L health dimension rather than calculate an overall utility score for study participants because we wanted to explore the heterogeneity in responses according to different aspects of health. Furthermore, as one of our main exposures of interest was migrant status, we were concerned about how well the current UK value set would be applicable to our study sample, which has a higher proportion of migrants than the general UK population.

The EQ-5D-5L VAS provides an alternative way to elicit an individual’s rating of their own overall current health. The respondent records their perception of their overall current health on a vertical visual analogue scale, where the endpoints are labelled ‘The best health you can imagine’ (100) and ‘The worst health you can imagine’ (0). The EQ-5D-5L VAS provides a quantitative measure of an individual’s perception of their overall health [21]. A full description and sample of the EQ-5D-5L is provided in the user manual [21].

### Exposures and covariates

We examined the relationship between sociodemographic factors including age, sex, Index of Multiple Deprivation (IMD) quintile, ethnicity, migration status and occupational group with EQ-5D-5L items across each of the five dimensions and the EQ-5D-5L VAS score.

IMD quintile was based on participants’ residential postcode data [22]. We asked participants to select their ethnicity from the 18 standardised groups used by the Office for National Statistics (ONS) in the 2021 Census for England and Wales. For analysis, the categories were collapsed into five aggregated ethnic groups (White, Asian, Black, Mixed and Other) used by the ONS. We used migrant status (whether the participant was born in the UK or overseas) to split each ethnic group in two, resulting in a ten-level categorical variable (see Table S1, Additional file 1: Supplementary Material). White UK-born was used as the reference group in regression models. For occupational group, participants were asked to select their main job/role from the following categories: medical, nursing, Allied Health Professional (AHP), pharmacy, healthcare scientist, ambulance, dental, optical, administrative and other (see Table S1 for further information).

We also collected data relating to health and lifestyle factors, which may mediate the effects of sociodemographic characteristics on outcome measures. These included number of self-reported long-term conditions (LTCs), smoking status, consumption of alcohol (units per week) and body mass index (BMI, using ethnicity-specific thresholds) [23]. A description of each variable and its derivation can be found in Table S1.

### Statistical analysis

We excluded respondents who did not provide information on outcome and exposure variables (i.e. EQ-5D-5L dimensions, EQ-5D-5L VAS, ethnicity and migration status) from the analysis. Categorical variables were summarised as frequencies and percentages, and non-normally distributed continuous covariates as the median and interquartile range (IQR). EQ-5D-5L VAS scores were non-normally distributed. Despite this, we presented plots of VAS scores by age as mean rather than median values, because of the tendency of scores to cluster around values of tens, and to a lesser extent, fives, as observed elsewhere [24]. EQ-5D-5L VAS scores have also been summarised using means and standard deviations in numerous other studies [25, 26]; our approach therefore facilitates comparison of our results with others. We plotted mean EQ-5D-5L VAS against age band to explore how associations between EQ-5D-5L VAS and other sociodemographic covariates changed by age, as this has been explored by other researchers [27].

We used logistic regression models to explore differences in reporting on the EQ-5D-5L dimensions by sociodemographic, occupational and health and lifestyle variables. We explored how each sociodemographic factor may mitigate the association of other sociodemographic factors by calculating/comparing three levels of odds ratio for the association between each sociodemographic factor and the EQ-5D-5L dimensions (in terms of likelihood of reporting any problems compared to no problems for each EQ-5D-5L dimension). We used univariable logistic regression to calculate unadjusted odds ratios (uORs) and multivariable logistic regression to calculate two sets of adjusted odds ratios (aORs), the first adjusting for all other sociodemographic variables and the second additionally adjusting for health and lifestyle factors likely to be on the causal pathway: alcohol and smoking consumption, BMI and presence of LTCs. Examining the change in aORs between models allows us to see how modifiable factors that may lie along the causal pathway to HRQoL may explain these observed patterns, using a sequential approach to addition of covariates, as used elsewhere [28]. As we formulated no a priori hypothesis, we have avoided overreliance on quoting absolute odds ratio values in favour of interpreting general trends.

We used linear regression models to explore differences in reporting EQ-5D-5L VAS scores by sociodemographic, occupational and health and lifestyle variables using the same three-level approach as used for the logistic regression analysis. We tested whether residuals were normally distributed after linear regression of EQ-5D-5L VAS scores by visually inspecting Q-Q plots. Although the resulting pattern indicated a left skewed distribution of the residuals, we elected to proceed with linear regression as our analysis method for EQ-5D-5L VAS as other studies have done [29, 30]. Linear regression is robust to variations in the normal residual assumption [31]. Importantly, violations of the assumption should not impact the coefficient estimates but could impact statistical tests for whether coefficients are equal to zero (again this is likely to be problematic only in studies with small sample sizes). We elected not to transform our outcome measure to improve the distribution of residuals because this is widely acknowledged to reduce the interpretability of results.

Multiple imputation was used to replace missing data in all logistic and linear regression models. Rubin’s Rules were applied to combine parameter estimates and standard errors from 10 imputed dataset into a single set of results [32]. Although indices of deprivation are available for UK countries outside England, these are not directly comparable with the English IMD [33]. We therefore elected to code IMD data as missing for those outside England and impute the missing information, as we have done previously [34]. Imputation models included all covariates used in the analysis as well as the exposures and the outcome of interest.

### Patient and public involvement

We worked closely with a Professional Expert Panel of HCWs from a range of ethnic backgrounds and occupations as well as with national and local organisations (see study protocol [11]).

### Role of the funding source

The funders had no role in the study design, data collection, data analysis, interpretation or writing of the report.

## Results

### Recruitment and formation of the study sample

The detailed formation of the study cohort is shown in Fig. S1 (Additional file 1: Supplementary Material). A total of 12,026 HCWs were included in this primary analysis.

### Description of the analysed study sample

Table 1 shows the sociodemographic, occupational and health characteristics of the analysed sample. The median age was 45 years (IQR: 34–54); excluding missing data in the denominator for each variable, most respondents were female (75.9%). 26.7% of respondents were overseas-born, and 29.9% were from non-White ethnic groups (19.2% Asian, 4.3% Black, 4.3% Mixed, 2.1% Other). The socioeconomic status of the study sample was higher than the UK population average, with half the sample (47.0%) residing in the least deprived areas (IMD quintiles 4 and 5) and only 8.7% in the most deprived quintile. AHPs were the largest group of participating HCWs by occupation, making up nearly a third of respondents (29.8%), followed by those in medical (22.9%) and nursing (20.0%) roles. The majority of respondents (50.7%) reported height and weight measurements which placed them in overweight or obese categories according to BMI. Most had never smoked (72.4%) and drank less than 14 units of alcohol/week (88.3%).

**Table 1:** Description of the UK-REACH analysed sample

Variable	Description, *n* (%) Total *n* = 12,026
Age in years, median (IQR)	45 (34–54)
Missing	65 (0.5)
Sex	
Male	2877 (23.9)
Female	9124 (75.9)
Missing	25 (0.2)
Ethnicity and migration status	
White UK-born	7388 (61.4)
White overseas-born	1043 (8.7)
Asian UK-born	850 (7.1)
Asian overseas-born	1464 (12.2)
Black UK-born	150 (1.3)
Black overseas-born	366 (3.0)
Mixed UK-born	381 (3.2)
Mixed overseas-born	128 (1.1)
Other UK-born	51 (0.4)
Other overseas-born	205 (1.7)
Occupational group	
Medical	2757 (22.9)
Nursing	2407 (20.0)
Allied Health Professional	3582 (29.8)
Pharmacy	235 (2.0)
Healthcare scientist	535 (4.5)
Ambulance	436 (3.6)
Dental	715 (6.0)
Optical	290 (2.4)
Administrative	236 (2.0)
Other	413 (3.4)
Missing	420 (3.5)
Index of Multiple Deprivation quintile	
1 (most deprived)	1041 (8.7)
2	1760 (14.6)
3	2184 (18.2)
4	2586 (21.5)
5 (least deprived)	3063 (25.5)
Missing	1392 (11.6)
Smoking status	
Never smoker	8708 (72.4)
Ex-smoker	2645 (22.0)
Current smoker	566 (4.7)
Missing	107 (0.9)
Weekly alcohol consumption	
None	4890 (40.7)
1–7 units	3865 (32.2)
8–14 units	1862 (15.5)
15–21 units	815 (6.8)
22–28 units	321 (2.7)
> 28 units	210 (1.8)
Missing	63 (0.5)
Number of long-term conditions, median (IQR)	0 (0–1)
Missing	508 (4.2)
BMI categories*	
Underweight	153 (1.3)
Healthy weight	4606 (38.3)
Overweight	3630 (30.2)
Obesity class 1 (BMI > 30)	1600 (13.3)
Obesity class 2 (BMI > 35)	569 (4.7)
Obesity class 3 (BMI > 40)	295 (2.5)
Missing	1173 (9.8)

*Using ethnicity-specific cut-offs (see Methods for details). All data are *n* (%) unless otherwise stated

*BMI* body mass index, *IQR* interquartile range

### EQ-5D-5L dimensions

**Fig. 1:**
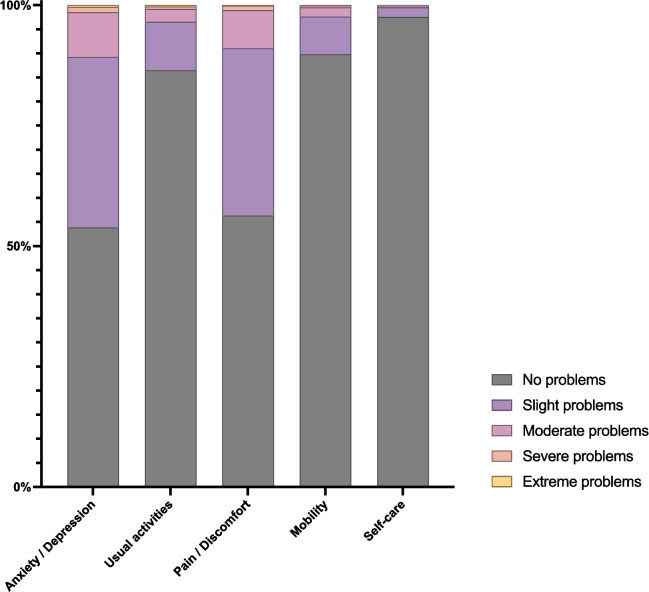
Breakdown of percentage of HCWs reporting level of problems for each of the five EQ-5D dimensions (anxiety/depression, usual activities, pain/discomfort, mobility and self-care)

The proportion of HCWs reporting any problems for each EQ-5D-5L dimension was 46.1% (anxiety/depression), 13.5% (usual activities), 43.7% (pain/discomfort), 10.2% (mobility) and 2.4% (self-care) (Table S2, Additional file 1: Supplementary Material). The breakdown of reporting severity of problems for each dimension is presented in Fig. 1. We found a low frequency of reporting severe and extreme problems in this working population, but also a relatively high occurrence of both slight and moderate problems with anxiety/depression and pain/discomfort. Compared to the Medical group, other HCWs groups reported more problems with pain/discomfort (e.g. prevalence of slight problems or more: 32.2% Medical, 55.4% Nursing, 45.4% AHPs, 49.8% Ambulance groups) and anxiety/depression (e.g. prevalence of slight problems or more: 36.8% Medical, 52.9% Nursing, 50.5% Ambulance groups).

**Fig. 2:**
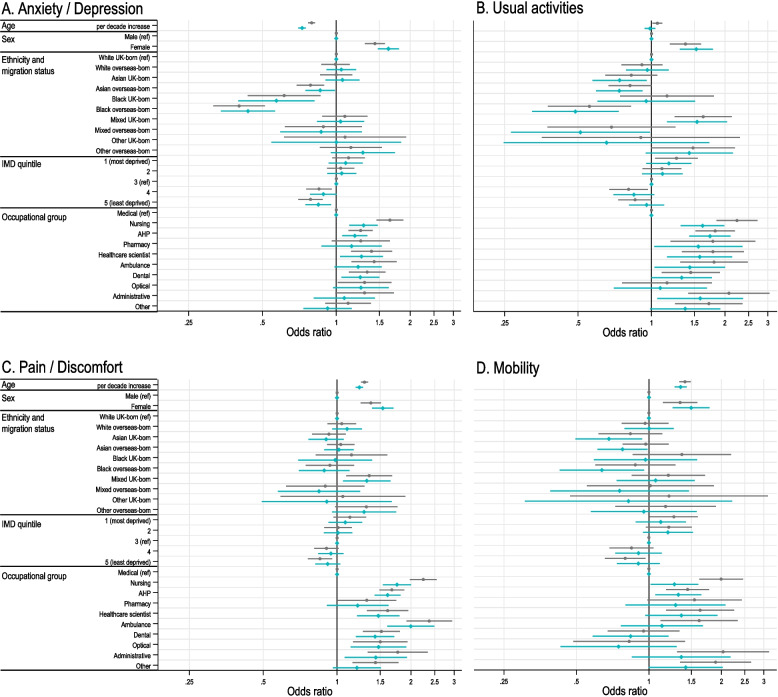
Results of multivariable logistic regression models showing the association of sociodemographic and occupational factors with reporting any problems on the individual EQ-5D-5L dimensions (pain/discomfort, mobility, usual activities, anxiety/depression), i.e. scoring more than 1 (no problems) on the 1–5 Likert score. Grey lines show models adjusting for all included sociodemographic variables (age, sex, ethnicity, migration status, IMD quintile) and occupational group. Green lines show models additionally adjusting for variables which may lie on the causal pathway (weekly alcohol consumption, smoking status, number of long-term conditions and BMI). Uncertainty bounds represent adjusted odds ratio 95% confidence intervals. AHP, Allied Health Professional; IMD, Index of Multiple Deprivation; ref, reference category. Results are shown for four of the five EQ-5D-5L dimensions and not shown for self-care due to the low scores reported for this dimension by this relatively healthy, active worker population. (Results for the self-care dimension and odds ratios for health factors are available on request. Unadjusted odds ratios are shown in Table S2, Additional file 1: Supplementary Material.)

Results of multivariable logistic regression models are shown in Fig. 2 (unadjusted odds ratios are shown in Table S2, Additional file 1: Supplementary Material). After adjustment for sociodemographic covariates, increasing age was associated with reporting more problems with pain/discomfort and mobility, but showed no association with problems undertaking usual activities, and older HCWs were significantly less likely to report anxiety/depression. Women were more likely than men to report problems across all studied dimensions. There was a consistent pattern of increased reporting of health problems with increased level of deprivation (indicated by IMD quintile) across EQ-5D-5L dimensions.

There were no consistent patterns of HRQoL by ethnic minority group and migration status across dimensions. Asian overseas-born and Black ethnic minority groups were less likely to report anxiety/depression compared with the White UK-born reference group. Black overseas-born HCWs were also less likely to report problems with usual activities but Mixed UK-born respondents were more likely to report problems for this dimension.

There were marked differences in reporting health problems by occupational group (Fig. 2; percentages reporting problems by severity shown in Fig. S2, Additional file 1: Supplementary Material). Nurses and ambulance workers appeared prominent in their higher frequency of reporting pain/discomfort (Fig. 2C), but generally all HCWs reported more problems with anxiety/depression, usual activities and pain/discomfort than the Medical group (Fig. 2A–C), with the pattern less clear for mobility (Fig. 2D).

Further adjustment for health and lifestyle variables attenuated the strength of associations observed for many of the covariates, for example, differences in reporting problems with each EQ-5D-5L dimension by IMD quintile. In contrast, odds of reporting problems on each EQ-5D-5L dimension for women actually increase once health and lifestyle factors are taken into account, and the same pattern is seen for age with the anxiety/depression dimension (Fig. 2A).

### EQ-5D-5L VAS scores

**Fig. 3:**
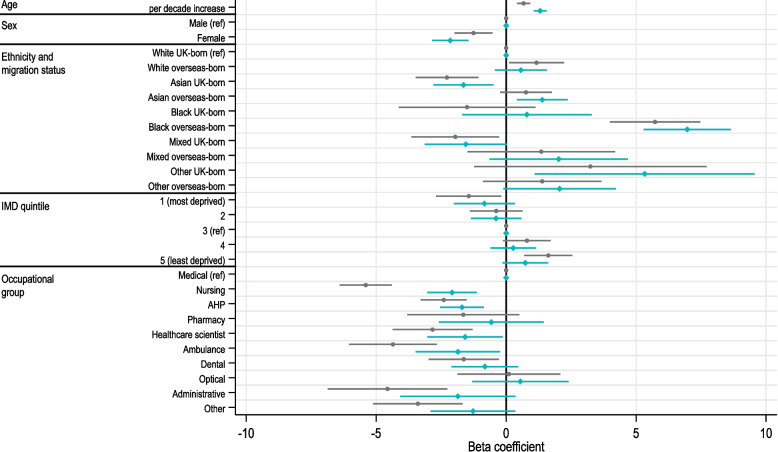
Multivariable linear regression model showing the association of sociodemographic, occupational and health and lifestyle factors with EQ-5D-5L visual analogue scores (VAS) where 100 represents ‘The best health you can imagine’ and 0 represents ‘The worst health you can imagine’. Grey lines show models adjusting for all included sociodemographic variables (age, sex, ethnicity, migration status, IMD quintile) and occupational group. Green lines show models additionally adjusting for variables which may lie on the causal pathway (weekly alcohol consumption, smoking status, number of long-term conditions and BMI). Uncertainty bounds represent beta coefficient 95% confidence intervals. Results of univariable linear regression models are shown in Table S3, Additional file 1: Supplementary Material. AHP, Allied Health Professional; BMI, body mass index; IMD, Index of Multiple Deprivation; LTC, long-term condition; ref, reference category

The mean (standard deviation) EQ-5D-5L VAS score of respondents was 77.8 (16.3). Linear regression models for EQ-5D-5L VAS scores are shown in Fig. 3. Increasing age was associated with a higher frequency of reporting health problems across EQ-5D-5L dimensions (Fig. 2), but conversely, it was also significantly associated with reporting higher EQ-5D-5L VAS scores, indicating higher assessment of HRQoL with age (Fig. 3). After adjusting for health and lifestyle factors (green line), this association became even stronger. Women reported lower HRQoL on EQ-5D-5L VAS score than men, a difference which again became more pronounced after adjusting for health and lifestyle factors. Black overseas-born HCWs reported substantially higher scores than most other groups ethnic/migrant groups.

There was a trend for increasing EQ-5D-5L VAS score with decreasing deprivation level (IMD quintile) and differences in EQ-5D-5L VAS by occupational group generally mirrored the reporting of EQ-5D-5L dimensions. Many HCW roles reported significantly lower EQ-5D-5L VAS scores than the Medical group, including Nursing, AHPs, Pharmacy, Healthcare scientists, Ambulance, Dental, Administrative and Other groups. Scores remained significantly lower, even after adjusting for health and lifestyle factors, for Nursing, AHPs, Healthcare scientists and Ambulance groups.

**Fig. 4:**
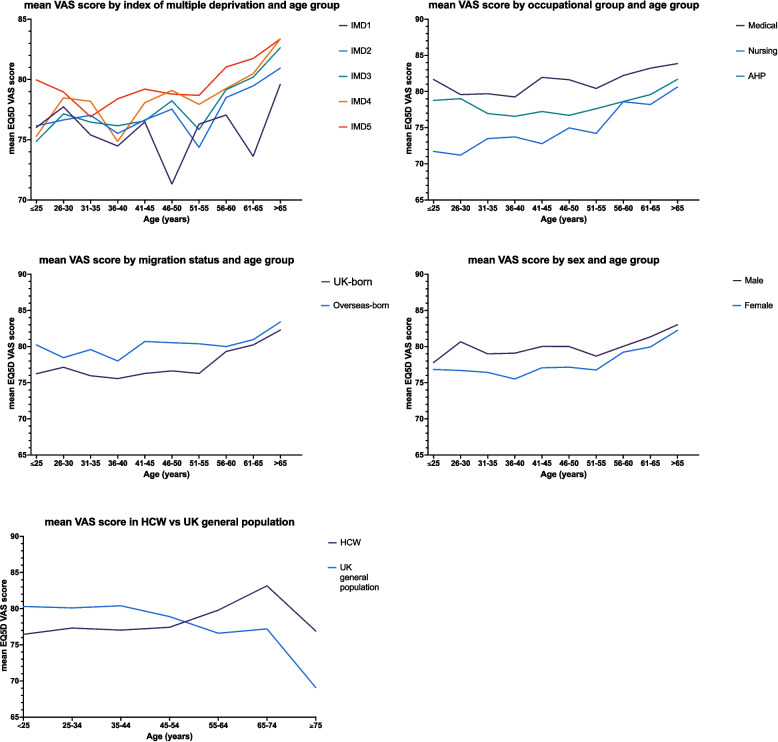
Mean EQ-5D-5L visual analogue scores (VAS) by age, stratified by **A** Index of Multiple Deprivation (IMD) quintile, 1—least deprived, 5—most deprived; **B** occupational group (medical, nursing, Allied Health Professional (AHP)); **C** migration status (UK- and overseas-born); and **D** sex. **E** compares scores with those from the UK general population, as reported by Janssen et al. [26] (note that the lowest age category for Janssen et al. is 18–24 years whereas for UK-REACH it is 16–24 years)

Figure 4 shows how HCWs’ EQ-5D-5L VAS scores vary by 5-year age band stratified by (A) IMD quintile, (B) the three largest occupational groups (Medical, Nursing, AHP), (C) migration status and (D) sex. All panels demonstrate the slight increase in EQ-5D-5L VAS with age. Figure 4A suggests a larger difference in EQ-5D-5L VAS scores by IMD quintile for those in the middle age groups (46–65 years). Differences in scores by occupational group may slightly reduce in older age groups (56 + years, Fig. 4B), a pattern which may also be seen when comparing UK-born to overseas-born respondents (Fig. 4C) and men with women (Fig. 4D).

## Discussion

We have reported findings from what is, to our knowledge, the largest study to evaluate HRQoL among HCWs. Our results provide a comprehensive overview of the self-reported physical and mental health of HCWs across the UK and may provide key insights to the BMJ commission on the future of the NHS, particularly regarding workforce sustainability and staff shortages. It is one of the first studies to explore differences in self-reported health of HCWs by occupation and other socioeconomic factors. We found that during the COVID-19 pandemic, HCWs reported high rates of anxiety/depression and pain/discomfort and their assessment of their HRQoL measured using the EQ-5D-5L VAS score was lower than that of the general UK population for ages up 45 years reported by another study. There appear to be substantial differences in HRQoL by occupation type and IMD quintile, with lower HRQoL associated with higher deprivation level. Medical staff reported higher HRQoL than other HCW occupations both on EQ-5D-5L VAS and frequency of reporting problems across the EQ-5D-5L dimensions.

The HCWs in our study reported EQ-5D-5L VAS scores that are lower than those reported elsewhere for the UK general population (Fig. 4E comparison with Janssen et al. [26]; mean EQ-5D-5L VAS 77.8 compared to mean 83.4 reported by EuroQual 2004, reference data for the UK [35]). While prevalence of reporting health problems was similar to UK population norms for mobility (UK-REACH 10.2% versus Janssen et al. [26] 12%), self-care (2.5% versus 4%) and usual activities (13.5% versus 11%), HCWs were far more likely to report problems with pain/discomfort (43.7% versus 28%) and anxiety/depression (46.1% versus 13%). Recent surveys have found high levels of mental health problems among UK HCWs, with NHS employees in two 2024 surveys reporting having had mental health issues (31% of more than 12,000 participants [36]) or a mental health condition (75% of 1078 participants [37]) in the past year. This issue is not restricted to the UK. The WHO-endorsed Bucharest Declaration on health and care workforce 2023 is a set of principles and priorities for improving the health workforce across Europe and Central Asia, which calls for the improvement of HCWs’ mental and physical health, acknowledging that many HCWs ‘continue to experience stress, burnout and violence, with some leaving their jobs’ [38]. A previous report produced by the WHO Regional Office for Europe similarly recognised the high levels of stress and burnout among HCWs across the region and their association with absenteeism and intention to leave the workforce [39].

Elevated anxiety/depression among HCWs has also been identified in a recent systematic review of other, smaller HCW studies conducted during the COVID-19 pandemic [40] and a narrative review focussing on nurses and midwives [41]; a study of 11,560 nurses in Spain found that over 80% reported negative psychological impacts due to the pandemic [42]. Public health emergencies such as the pandemic can exacerbate the already elevated levels of stress and mental health challenges experienced by HCWs, as well as presenting additional challenges in adapting quickly to changes in patient volume, mounting demands, clinical roles, new technologies and ways of working, uncertainties, elevated infection risk with limited protective equipment and managing the anxieties of patients and facing uncertainty in how to effectively treat and respond [43]. The pandemic may have impacted different HCW job roles differently, particularly for those in patient-facing roles [40, 44–48].

It has long been recognised that health is distributed unequally by occupation and socioeconomic status, with those lower on the occupational hierarchy having poorer health [49, 50]. In terms of such hierarchies within the health sector, previous, smaller studies from Europe have reported similar findings, with nurses reporting lower HRQoL than physicians [44, 51, 52]. We observed the same pattern for other HCW types, with lower psychological wellbeing and work-related quality of life compared to physicians reported for five occupations of the health and social care workforce (nurses, midwives, AHPs, social care workers and social workers) during the pandemic in the UK [53]. We also found high rates of pain reported by ambulance workers, which concurs with a recent review reporting high levels of back pain and back injuries among these staff due to lifting, working in awkward postures, loading patients into an ambulance and cardiopulmonary resuscitation procedures [54].

In contrast to normative data where self-reported EQ-5D-5L VAS scores decline with age (Janssen et al. [26]), in UK-REACH, mean scores increased with HCW age (Fig. 4E). This is likely due to sampling bias, as only working HCWs were included in our study population, who are likely to be relatively fit if they continue to work in HCW roles in the older age groups. In agreement with Janssen et al. [26], we observed that women tended to report more problems for all ages and all levels of deprivation across all EQ-5D-5L dimensions and these differences became more pronounced after adjusting for health and lifestyle factors. Similarly, European studies of healthcare workers undertaken during the COVID-19 pandemic found that women were at risk of higher psychological distress and worse quality of life during the first wave of the pandemic (Spain [55]) and reported significantly higher scores at secondary traumatisation related to their HCW role than men (Italy [56, 57]). Lower self-reported HRQoL by women has been widely reported [58–60], with the suggestion that women may perceive and react to health conditions differently from men [59–61].

There was no consistent pattern of differences in HRQoL comparing ethnic minority and migrant groups with White UK-born HCWs. Some groups reported markedly better HRQoL for certain metrics than White UK-born HCWs, particularly Black minority groups and Asian non-UK-born HCWs. The heterogeneity in our findings highlights differences in health outcomes of ethnic minority groups by migration status, as we have identified previously [62]. This demonstrates the importance of considering the intersection between ethnicity and migration status, particularly in HCWs, given the high proportion of workers from ethnic minority and migrant groups in the NHS [63, 64]. While there has been research undertaken exploring ethnic differences in HRQoL for conditions such as diabetes [65, 66], drawing conclusions is difficult because of inconsistencies in the measurement tools used and ethnic minority groups included [65]. In terms of work-related quality of life, studies of UK health and social care workers have found that respondents with Black ethnic background reported lower scores than White ethnicity respondents [67–69]. For the general population, Watkinson et al. reported the first study to document ethnic inequalities in HRQoL in a large, nationally representative English sample, which focussed on older adults (≥ 55 years) [70]. While study authors have criticised studies that aggregate distinct groups into broader categories because of small sample sizes, despite little or no similarity in health or exposures [70], similar to our findings, even with a large sample allowing for examination of outcomes for the smallest ethnic groups, estimates for some ethnic groups were imprecise.

Our multivariable regression analyses suggested that, for many of the sociodemographic categories and occupational groups investigated, elevated risks of poor HRQoL outcomes compared to the reference group were attenuated by additionally adjusting for health and lifestyle factors. This could reflect potential mediation by these factors. However, notable discrepancies to this pattern were observed for age (EQ-5D-5L VAS scores and EQ-5D-5L anxiety/depression dimension only) and sex (all EQ-5D-5L metrics). For age, this may represent an artefact of sampling bias of older HCWs remaining working in the healthcare system when other staff with lower HRQoL or anxiety/depression may have chosen to leave. There is a consistent pattern of female HCWs having greater odds of reporting lower HRQoL across all measures after adjustment for health and lifestyle factors than male workers. This suggests that lower HRQoL for female HCWs is unlikely solely to be a result of differences in health and lifestyle factors, such as elevated BMI or smoking, or facing a higher burden of long-term conditions.

There are some limitations to our study and analysis. Our present analysis focuses on HRQoL but not to more general aspects of wellbeing and quality of life, but these are the focus of other studies [44, 53], and for the UK, HCW studies have concentrated on wellbeing, stress, burnout and attrition rather than the full spectrum of physical and mental health effects [53, 71, 72]. The cross-sectional nature of these baseline data mean we can only comment on associations and not make causal inferences. However, the purpose of our analysis was hypothesis generation rather than proof of causation, for example, Asian overseas-born and Black ethnic minority groups having lower levels of anxiety/depression than White UK-born HCWs and nurses and ambulance workers more frequently experiencing pain/discomfort. Baseline UK-REACH data collection took place in 2020–2021 and therefore reflects HCWs’ opinions and experiences at the height of the first wave of the COVID-19 pandemic in the UK when HCWs’ workloads were substantially altered, increasing for some and decreasing for others, at a time of uncertainty and anxiety regarding infection risk [73]. HCWs’ circumstances at the time of questionnaire completion may have influenced their reporting of HRQoL, particularly regarding mental health. Despite the diversity of the study sample, we were unable to recruit large numbers from lower-banded occupations, which may limit the generalisability of our findings to those job roles. Our study of HCWs does not have a comparison group from the general population and so we have used data from an independent study for comparison [26]. Finally, there is inevitable selection bias within our self-selected cohort, which again may limit generalisability.

There are also a number of strengths of this analysis. The UK-REACH study sample is very large and diverse, ensuring adequate representation of this diverse workforce. While the majority of our study sample were women (75.9%), we nonetheless achieved a higher participation rate from the male workforce than other HCW studies (e.g. Neill et al., 88% women [53]), and the male to female ratio of respondents in our study is very similar to an estimate for the wider NHS workforce of 76.7% [74]. We achieved a high response rate from ethnic minority and migrant groups: 30% of our study sample were non-White; higher than the 26% in 2022 who were non-White across all NHS staff, for whom ethnicity was known [64] and higher than proportions achieved in most UK HCW studies (5% in Neill et al. [53]; not reported by Durkin et al. [71]).

## Conclusions

There is a moral imperative to prioritise and protect all aspects of HRQoL of the healthcare workforce. A high proportion of HCWs in our study reported problems with anxiety/depression and pain/discomfort compared to findings reported elsewhere for the general population. The threat to quality healthcare of these impacts must be minimised. Our findings can be used as a first step to understanding the underlying causes of lower HRQoL among HCWs, particularly in terms of anxiety/depression and pain/discomfort. Future research should pay particular attention to differences in HRQoL by occupational group and explore why women consistently report lower HRQoL. This better understanding of the causes of poorer health and quality of life among HCWs can we develop appropriate interventions to mitigate these effects and create a healthier, happier healthcare workforce.

## Supplementary Information

Additional file 1: Figures S1 and S2, Tables S1–S3. Fig. S1 Healthcare worker cohort recruitment flowchart. Fig. S2 Percentage of HCWs reporting level of problems for each of the five EQ-5D-5L dimensions stratified by occupational group. Table S1 Derivation of covariates from UK-REACH baseline questionnaire data. Table S2 Number of respondents reporting any problems on each of the four studied EQ-5D-5L dimensions stratified by sociodemographic and occupational factors, plus unadjusted odds ratios from univariable logistic regression models. Table S3 Mean EQ-5D-5L VAS scores and univariable linear regression models showing unadjusted beta coefficients for the association between sociodemographic, health and lifestyle factors with the EQ-5D-5L VAS score reported by each HCW.

## Data Availability

To access data or samples produced by the UK-REACH study, the working group representative must first submit a request to the Core Management Group by contacting the UK-REACH Project Manager in the first instance. For ancillary studies outside of the core deliverables, the Steering Committee will make final decisions once they have been approved by the Core Management Group. Decisions on granting the access to data/materials will be made within eight weeks. Third party requests from outside the Project will require explicit approval of the Steering Committee once approved by the Core Management Group. Note that should there be significant numbers of requests to access data and/or samples then a separate Data Access Committee will be convened to appraise requests in the first instance.

## Abbreviations

AHP: Allied Health Professional
aOR: Adjusted odds ratio
BMI: Body mass index
CHERRIES: Checklist for Reporting Results of Internet E-Surveys
EQ-5D: EuroQoL five-dimension
EQ-5D-5L: Five-level EQ-5D version
HCWs: Healthcare workers
HRQoL: Health-related quality of life
IMD: Index of Multiple Deprivation
IQR: Interquartile range
LTCs: Long-term conditions
ONS: Office for National Statistics
UK: United Kingdom
UK-REACH: United Kingdom Research study into Ethnicity and COVID-19 outcomes in Healthcare workers
uOR: Unadjusted odds ratio
VAS: Visual analogue scale
