# Supplementary material for: Sociodemographic factors associated with health-related quality of life in UK healthcare workers: a cross-sectional study
Source: BMC Med. 2025 Jul 22;23:438. doi: 10.1186/s12916-025-04208-6 (PMC12285068; doi:10.1186/s12916-025-04208-6)
Supplement: Supplementary file 1 — Additional file 1: Figures S1 and S2, Tables S1–S3. Fig. S1 Healthcare worker cohort recruitment flowchart. Fig. S2 Percentage of HCWs reporting level of problems for each of the five EQ-5D-5L dimensions stratified by occupational group. Table S1 Derivation of covariates from UK-REACH baseline questionnaire data. Table S2 Number of respondents reporting any problems on each of the four studied EQ-5D-5L dimensions stratified by sociodemographic and occupational factors, plus unadjusted odds ratios from univariable logistic regression models. Table S3 Mean EQ-5D-5L VAS scores and univariable linear regression models showing unadjusted beta coefficients for the association between sociodemographic, health and lifestyle factors with the EQ-5D-5L VAS score reported by each HCW. [file 12916_2025_4208_MOESM1_ESM.docx]

## **Supplementary Material**

**Sociodemographic factors associated with Health-related Quality of Life in UK healthcare workers:**

**a cross-sectional study**

Christopher A Martin (0000-0002-2337-4799), Clinical Fellow in Infectious Diseases^1,2,3,4¶^, Rebecca F Baggaley (0000-0002-4688-3924), Lecturer in Medical Statistics and Health Economics^2,4,5¶^, Lucy Teece, Lecturer in Medical Statistics^4,5^, Daniel Pan, NIHR Doctoral Research Fellow ^1,2,4,6,7^, Honorary Specialist Registrar in Infectious Diseases and General Internal Medicine(0000-0002-1268-2243)^3^, Joshua Nazareth (0000-0002-0603-9091), Academic Clinical Fellow in Infectious Diseases ^1,2,3,4^, Luke Bryant, Research Software Engineer (0000-0001-5231-4851)^1,4^, Carol Rivas, Professor of Health and Social Care^8^, Katherine Woolf (0000-0003-4915-0715), Professor of Medical Education Research^9^, Manish Pareek (0000-0003-1521-9964) Clinical Professor in Infectious Diseases.^1,2,3,4,10^ On behalf of the UK-REACH Study collaborative group.

1. Department of Respiratory Sciences, University of Leicester, Maurice Shock Medical Sciences Building, University Road, Leicester LE1 9HN, UK
2. Development Centre for Population Health, University of Leicester, Maurice Shock Medical Sciences Building, University Road, Leicester LE1 9HN, UK
3. Department of Infection and HIV Medicine, University Hospitals of Leicester NHS Trust, Leicester LE1 7RH, UK
4. NIHR Leicester Biomedical Research Centre (BRC), University of Leicester, George Davies Centre, 15 Lancaster Rd, Leicester LE1 7HA, UK
5. Department of Population Health Sciences, University of Leicester, George Davies Centre, 15 Lancaster Rd, Leicester LE1 7HA, UK
6. Li Ka Shing Centre for Health Information and Discovery, Oxford Big Data Institute, University of Oxford, Old Road Campus, Oxford OX3 7LF, UK
7. WHO Collaborating Centre for Infectious Disease Epidemiology and Control, School of Public Health, Li Ka Sing Faculty of Medicine, University of Hong Kong, Hong Kong, China
8. UCL Social Research Institute, University College London, 55-59 Gordon Square, WC1H 0NU, London, UK
9. UCL Medical School, University College London, 40 Bernard Street, Level 3, London WC1N 1LE, UK
10. NIHR Applied Research Collaboration East Midlands, University of Leicester, Leicester, UK

¶ Joint first authors.

Correspondence to: Professor Manish Pareek, Department of Respiratory Sciences, Maurice Shock Medical Sciences Building, University Road, Leicester LE1 9HN [manish.pareek@leicester.ac.uk](about:blank)

**Supplementary Methods**

UK-REACH study inclusion criteria: healthcare workers (HCWs) must be registered with one of the following seven UK professional healthcare regulatory bodies: the General Medical Council, Nursing and Midwifery Council, General Dental Council, Health and Care Professions Council, General Optical Council, General Pharmaceutical Council, or the Pharmaceutical Society of Northern Ireland.

## **Figure S1. Healthcare worker cohort recruitment flowchart.**

Healthcare workers included were those in professional healthcare roles or ancillary workers in a healthcare setting or registered with one of the seven participating UK healthcare professional regulatory bodies – see Martin et al^56^ for a list of participating regulatory bodies).

* There were 15,997 views of the questionnaire, 155 duplicate records were removed, leaving 15,842 unique HCW views.

† Corresponds to an effective response rate of 57.1% of those who registered/created a profile on the study website (and 84.5% of those who consented, 1.4% of those who were sent an email, and 3.2% of those who opened the email). HCW – healthcare worker.


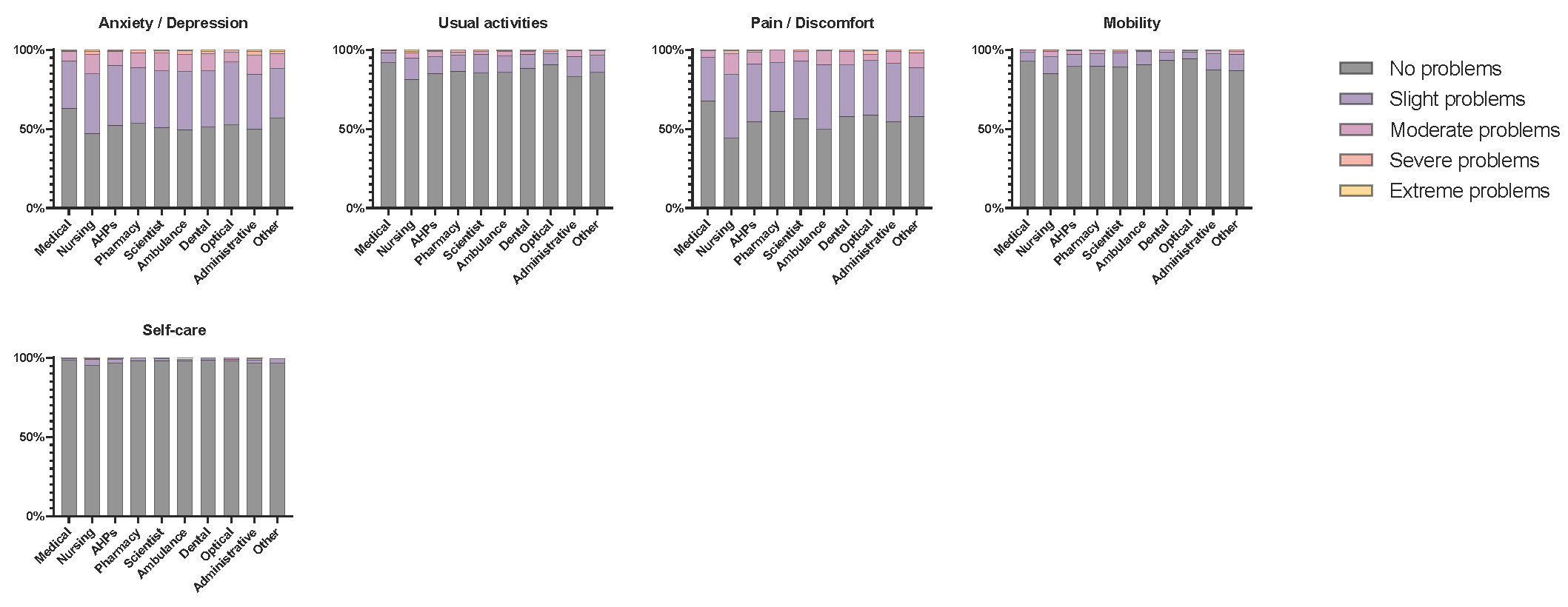


**Figure S2. Percentage of HCWs reporting level of problems for each of the five EQ-5D-5L dimensions (anxiety/depression, usual activities, pain/discomfort, mobility and self-care) stratified by occupational group.** AHP – Allied Health Professional.

## **Table S1** Derivation of covariates from UK-REACH baseline questionnaire data.

| **Variable** | **Description** |
| --- | --- |
| **Age** | Continuous variable. Age in years. Derived from date of birth entered by participants at registration. |
| **Sex** | Binary variable. Participants were asked their sex assigned at birth. |
| **Ethnicity** | Categorical variable. Participants were asked to select their ethnicity from a list of the 18 Office for National Statistics (O­­­NS) categories:  Asian/Asian British – Indian  Asian/Asian British – Pakistani  Asian/Asian British – Bangladeshi  Asian/Asian British – Chinese  Asian/Asian British – Any other Asian background  Black/African/Caribbean/Black British – African  Black/African/Caribbean/Black British – Caribbean  Black/African/Caribbean/Black British – Any other Black/African/Caribbean background Mixed/Multiple ethnic groups – White and Black Caribbean  Mixed/Multiple ethnic groups – White and Black African  Mixed/Multiple ethnic groups – White and Asian  Mixed/Multiple ethnic groups – Any other Mixed/multiple ethnic background  White – English/Welsh/Scottish/Northern Irish/British  White – Irish  White – Gypsy or Irish Traveller  White – Any other white background  Other ethnic group – Arab  Other ethnic group – Any other ethnic background  These were categorised into the 5 aggregated Office for National Statistics ethnicity categories (Asian, Black, Mixed, White, Other). |
| **Migration status** | Binary variable. Participants were asked whether they were born in the UK. |
| **Ethnicity and migration status combined** | Categorical variable. 18 ONS ethnicity categories reduced to five aggregated ethnicity categories (as also used by the ONS): White, Asian, Black, Mixed, Other, stratified by migration status (UK-born and overseas-born). |
| **Index of Multiple Deprivation (IMD) quintile** | Ordinal variable. Participants provided their residential postcode on registration for the study. This was used to determine the Index of Multiple Deprivation (the official measure of deprivation for small areas of England) in the area in which they live. The IMD ranks all areas in England based on 7 measures of deprivation and the ranks can be expressed as quintiles. Lower quintiles indicate more deprivation. Although Wales, Scotland and Northern Ireland have their own measures of deprivation, these are said not to be directly comparable to English IMD and therefore we elected to impute an ‘English IMD’ for residents of these nations.  **Source:** [https://www.gov.uk/government/statistics/english-indices-of-deprivation-2019](about:blank) |
| **Smoking status** | Binary variable. Participants were asked to indicate their current smoking status.  Three level categorical variable (never smoker, ex-smoker and current smoker). Never and ex-smokers were grouped together and compared with current smokers. |
| **Body Mass Index (BMI)** | Ordinal variable. Participants were asked to input their height and weight. These were used to calculate BMI in kg/m^2^. We then derived a 6-level ordinal variable using ethnicity-specific thresholds recommended by NICE: underweight, healthy weight, overweight, obesity class 1, obesity class 2 and obesity class 3.  **Source**: [https://www.nice.org.uk/guidance/cg189/chapter/Recommendations#identifying-and-assessing-overweight-obesity-and-central-adiposity](about:blank#identifying-and-assessing-overweight-obesity-and-central-adiposity) |
| **Alcohol use** | Ordinal variable. Participants were asked about their weekly consumption of alcohol in units selecting from the following categories: ‘none’, ‘1 to 7’, ‘8 to 14’, ‘15 to 21’, ‘22 to 28’, ’29 to 35’, 36 to 50’ and ‘≥51’.  Due to low numbers of participants reporting consumption of >21 units per week we collapsed these categories into one resulting in a five-level variable. |
| **Occupation** | Categorical variable. Participants were asked to select their main job/role. Categorised as below:  **Medical** – Doctor, Advanced Critical Care Practitioner, Anaesthesia associate, Surgical Care Practitioner, Other medical associate.  **Nursing** – Advanced Nurse Practitioner, Healthcare assistant, Maternity support worker, Midwife, Nurse, Nursing Associate, Other nursing and midwifery role.  **Allied Health Professional (AHP)** – Arts therapist, Chiropodist/Podiatrist, Dietician, Hearing aid dispenser, Occupational therapist, Operating department practitioner, Orthoptist, Physiotherapist, Practitioner psychologist, Prosthetist / Orthotist, Radiographer, Speech and language therapist, Occupational Therapy Support, Phlebotomist, Physiotherapy Assistant, Radiography, Other clinical support role, Other Allied Health Professional role.  **Pharmacy** – Pharmacist, Pharmacy technician, Other pharmacy role.  **Healthcare scientist** – Biomedical scientist, Clinical scientist.  **Ambulance** – Emergency medical, Paramedic, Other ambulance role.  **Dental** – Clinical dental technician, Dental Hygienist, Dental nurse, Dental technician, Dentist, Other dental role.  **Optical** – Dispensing optician, Optometrist, Other Optical role.  **Administrative** – Administration, Catering services, Domestic services, Estates services.  **Other** – Porter, Other. |
| **Long-term conditions** (Pre-existing health conditions) | Binary variables. Participants were asked to indicate if they had the following pre-existing health conditions:  1, Organ transplant  2, Diabetes (Type I or II)  3, Heart disease or heart problems  4, Hypertension  5, Overweight  6, Stroke  7, Kidney disease  8, Liver disease  9, Anaemia  10, Asthma  11, Other lung condition such as COPD, bronchitis or emphysema  12, Cancer  13, Condition affecting the brain and nerves (e.g., Dementia, Parkinson's, Multiple Sclerosis)  14, A weakened immune system or reduced ability to deal with infections (as a result of a disease or treatment)  15, Depression  16, Anxiety  17, Psychiatric disorders |
| **Long-term conditions (count)** | Continuous variable (count). We used information from the long-term conditions variable above to derive a variable that represented a count of the number of long-term conditions a participant reported. We did not include ‘overweight’, ‘anaemia’ or ‘psychiatric disorder’ in the count for reasons explained in the methods. |

**Table S2** Number (%) of respondents reporting any problems on each of the four studied EQ-5D-5L dimensions (pain/discomfort, mobility, usual activities, anxiety/depression, self-care) i.e. scoring at least 2 (slight problems) on the 1 (no problems) to 5 (extreme problems) likert scale stratified by sociodemographic and occupational factors, plus unadjusted odds ratios from univariable logistic regression models. Factors which are significant at the p<0.05 level are highlighted in bold. Results are not shown for the self-care EQ-5D-5L dimension due to the low scores reported for this dimension by this relatively healthy, active worker population but are available on request. 95%CI – 95% confidence intervals; AHP – Allied Health Professional; IMD – Index of Multiple Deprivation; IQR – interquartile range; med – median; ref – reference category; uOR – unadjusted odds ratio. Unadjusted odds ratios for the association between health factors and reporting any problems on the five EQ-5D-5L dimensions are also available on request.

| **Variable** | **Anxiety / Depression** | | **Usual activities** | | **Pain / Discomfort** | | **Mobility** | | |
| --- | --- | --- | --- | --- | --- | --- | --- | --- | --- |
|  | **N=5,544 (46.1)** | **uOR (95%CI)** | **N=1,622 (13.5)** | **uOR (95%CI)** | **N=5,252 (43.7)** | **uOR (95%CI)** | **N=1,221 (10.2)** | **uOR (95%CI)** |  |
|  |  |  |  |  |  |  |  |  |  |
| **Age,** med(IQR)* | **42 (33 – 52)** | **0.78 (0.76 – 0.81)** | 46 (35 – 54) | 1.04 (0.99 – 1.08) | **48 (37 – 55)** | **1.26 (1.23 – 1.30)** | **51 (40 – 57)** | **1.38 (1.31 – 1.45)** |  |
| **Sex**  Male  Female | 1,035 (36.0)  **4,500 (49.3)** | Ref  **1.73 (1.59 – 1.89)** | 271 (9.4)  **1,349 (14.8)** | Ref  **1.67 (1.45 – 1.92)** | 1,050 (36.5)  **4,185 (45.9)** | Ref  **1.47 (1.35 – 1.61)** | 228 (7.9)  **990 (10.9)** | Ref  **1.41 (1.21 – 1.64)** |  |
| **Ethnicity and migration status**  White UK-born  White overseas-born  Asian UK-born  Asian overseas-born  Black UK-born  Black overseas-born  Mixed UK-born  Mixed overseas-born  Other UK-born  Other overseas-born | 3,549 (48.0)  512 (49.1)  413 (48.6)  **545 (37.2)**  **56 (37.3)**  **100 (27.3)**  193 (50.7)  57 (44.5)  23 (45.1)  96 (46.8) | Ref  1.04 (0.92 – 1.19)  1.02 (0.89 – 1.18)  **0.64 (0.57 – 0.72)**  **0.64 (0.46 – 0.90)**  **0.41 (0.32 – 0.51)**  1.11 (0.90 – 1.36)  0.87 (0.61 – 1.23)  0.89 (0.51 – 1.55)  0.95 (0.72 – 1.25) | 1,092 (14.8)  137 (13.1)  **83 (9.8)**  **134 (9.2)**  25 (16.7)  **29 (7.9)**  **73 (19.2)**  12 (9.4)  5 (9.8)  32 (15.6) | Ref  0.87 (0.72 – 1.06)  **0.62 (0.49 – 0.79)**  **0.58 (0.48 – 0.70)**  1.15 (0.75 – 1.78)  **0.50 (0.34 – 0.73)**  **1.37 (1.05 – 1.78)**  0.60 (0.33 – 1.08)  0.63 (0.25 – 1.58)  1.07 (0.73 – 1.56) | 3,496 (46.1)  460 (44.1)  **288 (33.9)**  **554 (37.8)**  71 (47.3)  **141 (38.5)**  175 (45.9)  49 (38.3)  19 (37.3)  89 (43.4) | Ref  0.92 (0.81 – 1.05)  **0.60 (0.52 – 0.70)**  **0.71 (0.63 – 0.80)**  1.05 (0.76 – 1.45)  **0.73 (0.59 – 0.91)**  0.99 (0.81 – 1.22)  0.73 (0.51 – 1.04)  0.69 (0.39 – 1.23)  0.90 (0.68 – 1.19) | 822 (11.1)  99 (9.5)  **51 (6.0)**  **120 (8.2)**  22 (14.7)  32 (8.7)  38 (10.0)  12 (9.4)  5 (9.8)  20 (9.8) | Ref  0.84 (0.67 – 1.04)  **0.51 (0.38 – 0.68)**  **0.71 (0.58 – 0.87)**  1.37 (0.87 – 2.17)  0.77 (0.53 – 1.11)  0.88 (0.63 – 1.25)  0.83 (0.45 – 1.50)  0.87 (0.34 – 2.19)  0.86 (0.54 – 1.38) |  |
| **Index of Multiple Deprivation quintile**  1 (most deprived)  2  3  4  5 (least deprived) | **554 (53.2)**  890 (50.6)  1,058 (48.4)  **1,143 (44.2)**  **1,265 (41.3)** | **1.20 (1.03 – 1.40)**  1.10 (0.97 – 1.24)  Ref  **0.85 (0.75 – 0.95)**  **0.75 (0.67 – 0.84)** | **175 (16.8)**  263 (14.9)  303 (13.9)  **288 (11.1)**  **367 (12.0)** | **1.25 (1.03 – 1.52)**  1.08 (0.91 – 1.30)  Ref  **0.80 (0.66 – 0.95)**  **0.83 (0.71 – 0.98)** | 480 (46.1)  773 (43.9)  990 (45.3)  1,102 (42.6)  **1,281 (41.8)** | 1.04 (0.90 – 1.21)  0.95 (0.84 – 1.08)  Ref  0.90 (0.81 – 1.01)  **0.86 (0.77 – 0.96)** | 119 (11.4)  203 (11.5)  229 (10.5)  232 (9.0)  **269 (8.9)** | 1.14 (0.91 – 1.42)  1.11 (0.90 – 1.38)  Ref  0.85 (0.69 – 1.04)  **0.81 (0.67 – 0.98)** |  |
| **Occupational group**  Medical  Nursing  Allied Health Professional*  Pharmacy  Healthcare scientist  Ambulance  Dental  Optical  Administrative  Other | 1,015 (36.8)  **1,274 (52.9)**  **1,706 (47.6)**  **108 (46.0)**  **263 (49.2)**  **220 (50.5)**  **346 (48.4)**  **136 (46.9)**  **118 (50.0)**  **176 (42.6)** | Ref  **1.93 (1.72 – 2.15)**  **1.55 (1.41 – 1.72)**  **1.45 (1.10 – 1.89)**  **1.65 (1.37 – 1.98)**  **1.75 (1.43 – 2.14)**  **1.60 (1.36 – 1.89)**  **1.52 (1.19 – 1.94)**  **1.72 (1.32 – 2.24)**  **1.26 (1.02 – 1.55)** | 210 (7.6)  **446 (18.5)**  **535 (14.9)**  **32 (13.6)**  **76 (14.2)**  **60 (13.8)**  **84 (11.8)**  27 (9.3)  **39 (16.5)**  **57 (13.8)** | Ref  **2.74 (2.30 – 3.26)**  **2.12 (1.79 – 2.51)**  **1.91 (1.28 – 2.84)**  **2.00 (1.50 – 2.65)**  **1.92 (1.42 – 2.61)**  **1.61 (1.23 – 2.10)**  1.23 (0.81 – 1.87)  **2.46 (1.69 – 3.57)**  **1.94 (1.42 – 2.64)** | 887 (32.2)  **1,333 (55.4)**  **1,625 (45.4)**  **91 (38.7)**  **232 (43.4)**  **217 (49.8)**  **301 (42.1)**  **119 (41.0)**  **107 (45.3)**  **174 (42.1)** | Ref  **2.63 (2.35 – 2.94)**  **1.76 (1.59 – 1.95)**  **1.35 (1.03 – 1.77)**  **1.63 (1.36 – 1.97)**  **2.09 (1.71 – 2.57)**  **1.54 (1.30 – 1.82)**  **1.50 (1.17 – 1.92)**  **1.79 (1.36 – 2.35)**  **1.55 (1.26 – 1.91)** | 183 (6.6)  **360 (15.0)**  **357 (10.0)**  **24 (10.2)**  **57 (10.7)**  **41 (9.4)**  47 (6.6)  16 (5.5)  **30 (12.7)**  **54 (13.1)** | Ref  **2.50 (2.08 – 3.01)**  **1.57 (1.30 – 1.89)**  **1.61 (1.03 – 2.50)**  **1.70 (1.24 – 2.33)**  **1.47 (1.03 – 2.10)**  0.99 (0.71 – 1.39)  0.83 (0.49 – 1.40)  **2.13 (1.40 – 3.25)**  **2.15 (1.56 – 2.98)** |  |
| **Weekly alcohol consumption**  None  1 – 7 units  8 – 14 units  15 – 21 units  22 – 28 units  >28 units | 2,225 (45.5)  1,790 (46.3)  836 (44.9)  388 (47.6)  161 (50.2)  **110 (52.4)** | Ref  1.03 (0.95 – 1.13)  0.98 (0.88 – 1.09)  1.09 (0.94 – 1.26)  1.21 (0.96 – 1.51)  **1.32 (1.00 – 1.73)** | 749 (15.3)  **483 (12.5)**  **210 (11.3)**  **96 (11.8)**  45 (14.0)  31 (14.8) | Ref  **0.79 (0.70 – 0.89)**  **0.70 (0.60 – 0.83)**  **0.74 (0.59 – 0.92)**  0.90 (0.65 – 1.24)  0.95 (0.65 – 1.41) | 2,153 (44.0)  1,631 (42.2)  776 (41.7)  387 (47.5)  **169 (52.7)**  **111 (52.9)** | Ref  0.93 (0.85 – 1.01)  0.91 (0.82 – 1.01)  1.15 (0.99 – 1.34)  **1.42 (1.14 – 1.78)**  **1.43 (1.08 – 1.88)** | 614 (12.5)  **327 (8.5)**  **147 (7.9)**  **74 (9.1)**  **27 (8.4)**  27 (12.9) | Ref  **0.65 (0.56 – 0.74)**  **0.60 (0.50 – 0.72)**  **0.69 (0.54 – 0.90)**  **0.64 (0.43 – 0.96)**  1.03 (0.68 – 1.55) |  |
| **Smoking status**  Never smoker  Ex-smoker  Current smoker | 3,803 (43.7)  **1,343 (50.8)**  **340 (60.1)** | Ref  **1.33 (1.22 – 1.45)**  **1.94 (1.63 – 2.31)** | 1,075 (12.3)  **422 (16.0)**  **107 (18.9)** | Ref  **1.35 (1.19 – 1.52)**  **1.66 (1.33 – 2.07)** | 3,555 (40.8)  **1,366 (51.6)**  **285 (50.4)** | Ref  **1.54 (1.42 – 1.69)**  **1.47 (1.24 – 1.75)** | 793 (9.1)  **338 (12.8)**  **81 (14.3)** | Ref  **1.46 (1.28 – 1.67)**  **1.67 (1.31 – 2.14)** |  |
| **Number of long-term conditions, med(IQR)*** | **0 (1-1)** | **1.86 (1.76 – 1.95)** | **1 (0 – 2)** | **2.05 (1.93 – 2.17)** | **0 (1 – 1)** | **1.76 (1.67 – 1.85)** | **1 (0 – 2)** | **2.07 (1.94 – 2.20)** |  |
| **BMI category**†  Underweight  Healthy weight  Overweight  Obesity class 1 (BMI >30)  Obesity class 2 (BMI >35)  Obesity class 3 (BMI >40) | **83 (54.3)**  1,971 (42.8)  1,604 (44.2)  **777 (48.6)**  **329 (57.8)**  **185 (62.7)** | **1.49 (1.08 – 2.06)**  Ref  1.07 (0.98 – 1.17)  **1.27 (1.13 – 1.42)**  **1.79 (1.50 – 2.13)**  **2.19 (1.72 – 2.80)** | 17 (11.1)  452 (9.8)  **477 (13.1)**  **274 (17.1)**  **126 (22.1)**  **81 (27.5)** | 1.10 (0.67 – 1.81)  Ref  **1.39 (1.22 – 1.60)**  **1.90 (1.63 – 2.22)**  **2.63 (2.08 – 3.33)**  **3.41 (2.59 – 4.48)** | **38 (24.8)**  1,680 (36.5)  **1,627 (44.8)**  **817 (51.1)**  **327 (57.5)**  **202 (68.5)** | **0.58 (0.40 – 0.84)**  Ref  **1.41 (1.28 – 1.54)**  **1.85 (1.65 – 2.07)**  **2.36 (1.98 – 2.81)**  **3.65 (2.82 – 4.71)** | 9 (5.9)  252 (5.5)  **349 (9.6)**  **213 (13.3)**  **144 (25.3)**  **99 (33.6)** | 0.99 (0.49 – 1.98)  Ref  **1.85 (1.57 – 2.18)**  **2.77 (2.30 – 3.32)**  **5.73 (4.55 – 7.21)**  **8.46 (6.35 – 11.28)** |  |

uOR for each decade increase in age or each long-term condition.

† Using ethnicity-specific cut-offs (see Methods for details).

**Table S3** Mean (standard deviation) EQ-5D-5L VAS scores and univariable linear regression models showing unadjusted beta coefficients for the association between sociodemographic, health and lifestyle factors with the EQ-5D-5L VAS score reported by each HCW. 95%CI – 95% confidence intervals; AHP – Allied Health Professional; BMI – body mass index; IMD – Index of Multiple Deprivation; LTC – long-term condition; ref – reference category; sd – standard deviation.

| **Variable** | **Mean (sd)**  **Total n = 12,026** | **Beta coefficient, (95%CI)** |
| --- | --- | --- |
| **Age (years)**  <30  30 – 39  40 – 49  50 – 59  ≥60 | 77.1 (16.5)  76.9 (16.8)  77.7 (16.4)  77.9 (16.6)  81.1 (14.3) | 0.82 (0.58, 1.07)* |
| **Sex**  Male  Female | 79.9 (15.3)  77.2 (16.7) | Ref  -2.72 (-3.41, -2.04) |
| **Ethnicity and migration status**  White UK-born  White overseas-born  Asian UK-born  Asian overseas-born  Black UK-born  Black overseas-born  Mixed UK-born  Mixed overseas-born  Other UK-born  Other overseas-born | 77.3 (16.6)  78.5 (15.5)  76.1 (16.9)  80.0 (16.1)  75.8 (18.2)  83.5 (13.6)  75.9 (16.3)  79.4 (16.0)  82.3 (15.4)  80.3 (16.3) | Ref  1.24 (0.18, 2.30)  -1.16 (-2.32, 0.00)  2.72 (1.80, 3.64)  -1.47 (-4.11, 1.17)  6.24 (4.53, 8.00)  -1.33 (-3.01, 0.35)  2.11 (-0.75, 4.97)  5.08 (0.57, 9.58)  3.06 (0.79, 5.33) |
| **IMD quintile**  1 (most deprived)  2  3  4  5 (least deprived) | 75.6 (18.3)  76.8 (17.5)  77.3 (16.4)  78.2 (15.7)  79.3 (15.6) | -1.78 (-3.04, -0.52)  -0.61 (-1.63, 0.41)  Ref  0.88 (-0.04, 1.80)  1.97 (1.05, 2.89) |
| **Occupational group**  Medical  Nursing  AHP  Pharmacy  Healthcare scientist  Ambulance  Dental  Optical  Administrative  Other | 81.0 (14.4)  74.9 (18.1)  77.7 (16.0)  78.5 (15.5)  77.4 (15.5)  75.8 (17.7)  78.5 (16.2)  80.2 (15.2)  75.3 (18.0)  77.1 (17.3) | Ref  -6.06 (-7.0, -5.16)  -3.21 (-4.01, -2.40)  -2.52 (-4.70, -0.34)  -3.54 (-5.05, -2.04)  -5.15 (-6.79, -3.50)  -2.45 (-3.78, -1.11)  -0.70 (-2.67. 1.26)  -5.83 (-8.08, -3.59)  -3.88 (-5.57, -2.19) |
| **Alcohol consumption**  None  1 – 7 units  8 – 14 units  15 – 21 units  22 – 28 units  >28 units | 77.0 (17.7)  78.5 (15.6)  79.7 (14.0)  78.2 (15.2)  76.4 (16.8)  72.7 (18.8) | Ref  1.48 (0.78, 2.17)  2.72 (1.84, 3.60)  1.22 (0.01, 2.44)  -0.58 (-2.42, 1.27)  -4.18 (-6.44, -1.92) |
| **Smoking status**  Never smoked  Ex-smoker  Current smoker | 78.6 (16.1)  76.3 (16.7)  73.9 (18.7) | Ref  -2.22 (-2.93, -1.51)  -4.67 (-6.07, -3.25) |
| **Number of long-term conditions**  0  1  2  ≥3 | 81.5 (13.6)  74.3 (17.7)  69.0 (19.5)  63.0 (22.0) | -6.06 (-6.41, -5.70)* |
| **BMI categories**†  Underweight  Healthy weight  Overweight  Obesity class 1 (BMI >30)  Obesity class 2 (BMI >35)  Obesity class 3 (BMI >40) | 80.6 (14.8)  81.5 (14.1)  78.4 (15.5)  73.7 (18.4)  69.8 (18.6)  65.8 (20.0) | -0.48 (-2.99, 2.02)  Ref  -3.19 (-3.88, -2.51)  -7.90 (-8.84, -6.97)  -11.62 (-13.11, -10.13)  -15.62 (-17.69, -13.55) |

* Beta coefficient for each decade increase in age or each long-term condition.

† Using ethnicity-specific cut-offs (see Methods for details).
